# Mediation effects of post-series depression on the relationship between life satisfaction and positive mental health of Vietnamese: A cross-sectional study in COVID-19 pandemic context

**DOI:** 10.3389/fpsyg.2022.971711

**Published:** 2022-11-28

**Authors:** Be Thi Ngoc Nguyen, Son Van Huynh, Trong Nguyen Nguyen, Bao-Tran Nguyen-Duong, Thuy-Trinh Ngo-Thi, Vinh-Long Tran-Chi

**Affiliations:** ^1^Department of Psychology and Education, University of Education, Hu´ ê University, Hu´ ê, Thua Thien Hue, Vietnam; ^2^Faculty of Psychology, Ho Chi Minh City University of Education, Ho Chi Minh City, Vietnam; ^3^Scientific Management Department, Dong A University, Da Nang, Vietnam

**Keywords:** positive mental health, life satisfaction, post-series depression, mediation, PLS-SEM

## Abstract

Vietnam, a middle-income country, has been suffering four waves of the Coronavirus disease 2019 (COVID-19) pandemic and a massive lockdown to suppress the spread of this infectious disease. Consequently, COVID-19 has caused psychological ramifications and affected humankind’s life satisfaction. Because of the lockdown period, numerous people had plentiful time. Hence, they found solace in excessive watching of television and movies, which could lead to post-series depression. The purpose of this study is to investigate the relationship between life satisfaction (LS), post-series depression (PSD), and positive mental health (PMH) and inquire about the mediation effect of satisfaction of life and PSD. A total of 2,572 participants who were voluntarily recruited from various media platforms completed self-report questionnaires, including the Satisfaction with life scale, Post-series depression scale, and Positive Mental Health Scale. This study was assessed using the PLS-SEM approach. The findings of this research discovered (i) a significantly positive effect of LS on PMH; (ii) a significantly negative effect of PSD on PMH; (iii) a significantly negative effect of LS on PSD, and (iv) a significant indirect effect of LS on PMH through PSD. The study provided additional evidence to the relationship between life satisfaction and PMH of individuals. Besides, the negative effects of PSD, which is a non-clinical term for feeling down that frequently appears after individuals finish their much-loved film and TV series, on individuals’ PMH is proved, especially in the COVID-19 pandemic context in which Vietnamese people must remain in their current location.

## Introduction

Since the termination of World War II, Coronavirus disease 2019 (COVID-19) emerged in December 2019 in Wuhan, China, and has become one of the most threats to humanity in 75 years. The spread of this infectious disease has been widening every single day. Owing to this, the effects of this virus are populated in all areas of life. There is a need to implement appropriate mental health measures and physical health precautions, especially in undeveloped nations, as the coronavirus pandemic has disseminated dread on an individual level but at a social level (Abbas, 2020). Besides medical tests and treatments, many nations applied temporary restrictions, including social distancing and cancelation of gatherings and meetings. Several nations have imposed severe restrictions on school attendance, working from home, quarantine with numerous cases, and, most crucially, lockdowns to decrease COVID-19 cases. Like most nations, Vietnam also had a palpable effect on the COVID-19 pandemic. Owing to this impact, the government of Vietnam decided to conduct a mandatory nationwide lockdown in which residents were required to stay at home and could only leave for “essential needs.” This strict measurement lasted more than three months with 28 cities and metropolises.

These measurements have demonstrated their usefulness in suppressing the spread of this infectious disease; despite this fact, they also have unintended consequences for various aspects of citizens’ lives. The epidemic exacerbated mental health difficulties and various forms of human suffering (Vindegaard and Benros, 2020). Moreover, a widespread lockdown also had psychological ramifications. People endure increased anxiety and sadness due to social distancing and closure (Cellini et al., 2020). With a lasting lockdown, limitations on public life, and job security, psychological illnesses such as worry, stress, and depression may be on the rise among the population (Shi et al., 2020; Rehman et al., 2021). Furthermore, with the lockdown, people have a great deal of time to access numerous social media platforms and information on the Internet. Consequently, people may have negative psychological impacts due to media reports containing infodemics about COVID-19’s impact on mental health (Su et al., 2021).

By dint of spreading this pandemic, studying, working, and entertaining during the lockdown need to be taken through the Internet. Due to the closure of schools with in-person learners, online learning has been strengthened, and working at home has boosted Internet usage among children and teenagers (Ngamije, 2021). Much related research has found that lockdown has a considerable negative impact on university students’ daily social connections and social support. In addition to the ongoing stressors connected to the epidemic, these have the potential to significantly impact students’ mental health (Aqeel et al., 2022). Besides that, many forms of leisure and entertainment have collapsed, including restaurants, movie theaters, parks, and sports clubs.

Consequently, the entertainment industry is now exclusively dominated by the Internet (Thakur et al., 2020). Against this backdrop, Király et al. (2020) reported that for most people, Internet usage helps to reduce loneliness and alleviate negative moods. Therefore, people during mandatory lockdown time usually watch Tiktok, a cooking instructions series to improve their skills on Youtube; moreover, Netflix, with its variety of web and movie series, is the most chosen option for them to binge-watch (Thakur et al., 2020). Concomitantly, Farber et al. (2020) assumed that watching movies and television could help many individuals reduce a great deal of pressure during these times. Because of the influence of media entertainment, specifically watching movies or TV series, on the mental health of Vietnamese people at the time of lockdown, the authors conducted a study on a group of Vietnamese people who had full awareness and feelings about the movies in the most comprehensive way.

### Post-series depression

Post-series depression (PSD) is a recently developed concept. Although there is a lack of academic research and definition based on gray literature, also called fugitive literature, PSD still has great application potential regarding psychological context. From the original paper, PSD is a context-specific mood state, a term used to express the feelings of emptiness, loss, and melancholy of arts and leisure lovers after their favorite screen products, such as movies and TV series, were discontinued (Kottasz et al., 2019). These special feelings for a screen product can result from the fans’ parasocial relationship with TV stars (Cohen, 2004). PSD is also considered a non-clinical term for feeling down, frequently appearing when individuals finish their much-loved film and TV series (Regine, 2015).

The emotions that are manifestations of PSD (e.g., emptiness, melancholia) can negatively affect individuals’ mental health. Emptiness could make people want to “fill up something missing inside” by excessive use of an addictive substance, television addiction, excessive computer use or internet access, or compulsive use of video or computer games that would lead to significant impairment to the abilities to function effectively in daily life (Kottasz et al., 2019). The concept of melancholia comprises mood disturbance, disturbance in cognition or behavior, and relates to a depressed mood (Tondo et al., 2020). Thus, PSD could be an indirect measurement for predicting the mental health of individuals engrossed with screen products. However, the construct should be considered a brief mood state rather than a clinical depression (Regine, 2015).

Moreover, PSD is discussed to correlate with other negative emotions. Kottasz et al. (2019) concluded that individuals with feelings of nostalgia and emptiness were significantly more likely to experience PSD than others. Nostalgia is a mixed emotion (Newman and Sachs, 2020) that happens when a person is craving sentimentally for the past (Cheung et al., 2013) or when they believe that their past was better than their recent life found that nostalgia, along with loneliness was negatively affecting wellbeing. It exaggerated the negative effects of loneliness on affective wellbeing (Newman and Sachs, 2020). Besides, Karlin (2015) mentioned several emotions directly related to PSD as “a sadness felt after watching a long series or story. The bitter feeling when you know the journey is over, but you don’t want it to end. All you know is that your stomach drops when the episode cuts to credits.” The more deeply people are involved with the narrative, plot, actors’ performances, and screen characters in movies or screen series, the more negative the feelings triggered in those people when the screen products are terminated. Among A&L fandom, PSD was found to be positively correlated with binge-watching (Kottasz et al., 2019) and underlying compulsive buying behavior of screen-related products (Kottasz et al., 2019; Kottasz and Bennett, 2020). Binge-watching can be caused by individuals’ feelings of emptiness and disappointment because of an unsatisfied ending or the loss of a much-loved screen experience after a sustained involvement with a favorite screen product (Jones et al., 2018). Overall, PSD has a strong negative effect on the mental health of screen products’ fans.

### Life satisfaction

Because of the palpable effects of the COVID-19 pandemic, the government of Vietnam enforced a strict COVID lockdown which has affected people’s daily lives and mental health by the length and difficulty of being confined at home, fears of infection, frustration, boredom, insufficient supplies and information, and financial loss (Webster et al., 2020). The COVID-19 epidemic has had a negative effect on the economy, and individuals have lost their jobs, failing to achieve a minimum income level to support their standard of living (Yoosefi Lebni et al., 2021). Fear of economic loss has made individuals more stressed and contributed to mental health issues on a worldwide scale (Yoosefi Lebni et al., 2021). The levels of life satisfaction are influenced by socioeconomic status (Bellis et al., 2012), employment status (Dolan et al., 2008), financial resources (Van Praag et al., 2003), intimate relationships, physical health, personality and community involvement (Dolan et al., 2008). Therefore, the negative effects of social restrictions and physical distancing could lead to the reduction of people’s life satisfaction and then cause mental health problems.

Life satisfaction has been considered an important and interesting research topic to study extensively since the late 20th century (Kim et al., 2021). Generally, life satisfaction is the individuals’ sense of overall happiness with life (Lent et al., 2005), the appraisal of individuals’ current life conditions (Diener and Suh, 2000) based on people’s evaluation criteria of material living conditions, physical and mental health, education, social relationship, natural environment, economic security (World Health Organization [WHO], 1996). Life satisfaction protects adolescents from negative events and mental health problems by mitigating severe effects Field (Suldo and Huebner, 2004) and plays an important psychological factor in the process that good-quality relationships improve individuals’ mental health (Cavioni et al., 2021). Life satisfaction is characterized by several positive emotions, such as happiness (Hofmann et al., 2014), which is one of the most desirable goals in people’s life. Cloninger and Zohar (2011) indicated that human happiness could arise from positive feelings and the reduction of negative feelings, which could promote individuals’ mental health. Several authors suggested that life satisfaction was negatively associated with a negative affective state, leading to mental health problems such as psychological distress, depression, and anxiety (Marum et al., 2014; Beutel et al., 2016). The term life satisfaction is not the superordinate concept of mental health. Still, it is an important indicator that should be investigated and examined when studying aspects of the positive mental health (PMH) (Kjell et al., 2016).

### Positive mental health

Positive mental health, generally called mental wellbeing, is an important factor that should be assessed to ensure individuals’ mental health (Lukat et al., 2016). Barry (2009) claimed that PMH is a key asset and resource for the population’s wellbeing and society’s long-term social and economic prosperity. The conception of PMH encompasses all the senses of fulfillment about emotion (affect/feeling), psychology (positive functioning), society (relations with others and society), physic (physical health), and spiritual beliefs (sense of meaning and purpose in life) (Barry, 2009). This definition of PMH comes from two distinct perspectives of wellbeing, namely hedonic and eudaimonic (Keyes, 2002). While hedonic represents the will of personal happiness (Ryan and Deci, 2001) in the existing termination, including emotional, psychological, and spiritual wellbeing, the eudaimonic illustrates the purpose of life (Ryan and Deci, 2001). From the above, Lukat et al. (2016) suggest that the concept involving emotional, psychological, and social wellbeing is PMH. Therefore, PMH can be conceptualized as a positive emotion encompassing a sense of coherence, subjective wellbeing, optimism, life satisfaction, and personality traits, including self-realization, autonomy, and resilience when confronting stressors, coping with adversity, and avoiding breakdown (World Health Organization [WHO], 2004). During COVID-19, having high PMH has become more important than ever owing to its psychological resilience ability and prevention of mental illness. It’s a protective factor (Lukat et al., 2016) which not only is the most important predictor of remission from anxiety disorders, specific phobia (Trumpf et al., 2010), suicidal thoughts (Teismann et al., 2016) but also precludes effect of depressive symptoms on suicide ideation (Teismann et al., 2018).

Positive mental health is a complex concept, so knowing which is the determining factor can rarely be straightforward. Factors influencing PMH come from biological, psychological, social, economic, and environmental sources. However, to the best of our knowledge, there is limited research on factors influencing individuals’ PMH, especially in the Vietnamese context. This country had a palpable effect on the COVID-19 pandemic. In the present study, we aim to examine the direct and indirect effects of life satisfaction and PSD on individuals’ PMH. We expected the relationship between factors to be reciprocal, that life satisfaction had a significant and positive effect on PMH, but it would negatively affect the level of PSD. Moreover, we also expected there was a partial meditation role of LS or PSD. Barry (2009) suggested that more study should focus on the “web of causation” between the above factors; therefore, the LS-SEM approach in our study is justifiable.

## Materials and methods

### Research hypotheses

Our cross-sectional study investigates the relationship between PSD, LS, and PMH among Vietnamese university students.

**Hypothesis 1:** A high level of life satisfaction would promote individuals’ PMH.

**Hypothesis 2:** Individuals with a high level of life satisfaction would be less likely to experience PSD.

**Hypothesis 3:** Experiencing PSD would negatively affect individuals’ PMH.

**Hypothesis 4:** Age would moderate the relationship between life satisfaction and PMH.

**Hypothesis 5:** Post-series depression would mediate the relationship between life satisfaction and PMH.

**Hypothesis 6:** Post-series depression would mediate the relationship between demographic characteristics and PMH.

**Hypothesis 7:** Life satisfaction would mediate the relationship between demographic characteristics and PMH.

### Participants

Subjects were voluntarily recruited from several universities, institutions, and social media platforms. The participant’s sample frames were collected from two metropolises and two provinces in Vietnam. A total of 2,909 questionnaire surveys were delivered, all of which were returned. After the elimination process, 337 responses were rejected due to insufficient information loss of fidelity as the same answers for all questions; therefore, the last sample was 2,572. The data set involved 591 males (23.0%) and 1981 females (77.0%), in which high school participants were 692 (26.9%), undergraduate participants were 1,547 (60.1%), and postgraduate participants were 333 (12.9%); with 680 participants (26.9%) aged 15–18, 1,387 at the age of 19–22 years and the rest are aged 23 to under 30 years old (*n* = 505). In Table 1, age groups of 18–22 and over 22 are respectively understood as age groups of 19–22 and 23–30. The table illustrates the descriptive statistics of participants with the effect of demographic characteristics on PMH, Life satisfaction, and PSD.

**TABLE 1:** Sample descriptive characteristics.

	Total (*n* = 2572)	PSD		PMH		LS	
	Frequency	Mean ± SD	*p*	Mean ± SD	*p*	Mean ± SD	*p*
Age, year	–	–	< 0.001	–	< 0.01	–	0.994
15–18	680 (26.4)	2.69 ± 0.63	–	3.52 ± 0.66	–	3.28 ± 0.82	–
18–22	1387 (53.9)	2.78 ± 0.67	–	3.51 ± 0.65	–	3.28 ± 0.86	–
Over 22	505 (19.6)	2.55 ± 0.7	–	3.63 ± 0.71	–	3.28 ± 0.9	–
Gender	–	–	< 0.001	–	< 0.01	–	< 0.001
Men	591 (23.0)	2.62 ± 0.65	–	3.67 ± 0.74	–	3.09 ± 0.91	–
Women	1981 (77.0)	2.74 ± 0.67	–	3.56 ± 0.64	–	3.33 ± 0.83	–
Education	–	–	< 0.001	–	0.139	–	0.984
High school	692 (26.9)	2.68 ± 0.63	–	3.53 ± 0.66	–	3.28 ± 0.82	–
University	1547 (60.1)	2.76 ± 0.67	–	3.53 ± 0.66	–	3.28 ± 0.86	–
Postgraduate	333 (12.9)	3.56 ± 0.72	–	3.61 ± 0.71	–	3.27 ± 0.91	–
Total	–	2.71 ± 0.67	–	–	–	–	–

PSD, post-series depression; LS, life satisfaction; PMH, positive mental health.

### Instruments and procedures

#### Instruments

The PSD, stands for The PSD, was developed by Kottasz et al. (2019) to examine the sense of loss, desolation, and melancholy occurring when a movie or TV series ends. This scale had 15 self-report items (e.g., After the termination of the TV/film series, I felt Disappointed.), with one factor after the authors conducted a principal components exploratory factor analysis and eliminated nine items. All items were rated on a five-point Likert scale ranging from 1 = Never to 5 = Always. In the original paper, a Confirmatory Factor Analysis was followed by the item development process to indicate a robust 1-factor solution; the model fit in the original scale was χ^2^/df = 2.2, CFI = 0.97, GFI = 0.94, TLI = 0.91, RMSEA = 0.05. Factor loading of all 15 items ranged from 0.702 to 0.842 (*t* < 0.01), which satisfied the elimination criteria (< 0.5) (Hair et al., 2019), with the Average Variance Extraction (AVE) of 0.7 showed that all items considerably explained the variance of the theory construct (AVE > 0.05) (Fornell and Larcker, 1981). Additionally, the Cronbach’s α for the 15 items was 0.88; composite reliability = 0.77.

Life satisfaction: the Satisfaction with Life scale (Diener et al., 1985) was developed to measure global satisfaction with life rather than with specific aspects of life (e.g., finance, family,…). The scale has five items, responding from 0 (Never) to 5 (Always), and has favorable psychometric properties (Diener et al., 1985).

Positive mental health: the PMH scale is a well-developed assessment (Lukat et al., 2016) measuring the inner and outer factors associated with mental wellbeing (i.e., emotional, psychological, and social aspects of wellbeing). The scale involved nine person-centered self-reported items (e.g., In general, I am confident) rating on a Likert scale from 1 (not true) to 4 (true) is unidimensionally constructed, brief, easy to complete, and sensitive to change.

#### Procedures

Informed consent was obtained prior to the enrollment of subjects; participants’ obligations and right to withdraw from the study were detailed in the information sheet. Therefore, they could withdraw from the study if they could not continue. About the questionnaires, the subjects were acquainted with the aim of this research and asked to provide their information, including gender, education, and residence location. The participants completed their self-report information and questionnaires under the guideline and insurance of research instructors. These participants did the survey through a link through Google Form, which the authors provided during the final lockdown periods in Vietnam. The data collection took three weeks as part of a 4 month research project, from September 2021 to December 2021, which was the last phase of the lockdown period and the first month after the Vietnamese government’s lockdown was lifted.

The translation process of the 15-item PSD Scale, 5-item satisfaction with life scale (SWLS), and 9-item PMH Scale was conducted according to the translation and back-translation process guidelines. First, we asked the authors of the PSD Scale for permission to translate and validate this scale for the Vietnamese population. Besides, SWLS and PMH Scale have been used publicly and widely. Consequently, the authors did not need to ask permission to use them. Then, we recruited a Vietnamese native speaker, who is fluent in English, comprehends cultures, and has experience in translating exercises to help our translation.

A Vietnamese translator generated a forward version of the scale. Following that, all research members agreed to reconcile the initial Vietnamese translation to provide an adequate ultimate translation for backward translation. The back-translation from Vietnamese translation to English was undertaken by an English translator who speaks English as a native language and is fluent in Vietnamese. After receiving the back-translation, the research group compared it with the original scale to ascertain whether there were any contradictions or discrepancies. After examining the scale’s application, no problems were discovered, and the final Vietnamese version of the PSD Scale, SWLS, and PMH Scale was formally accepted to use.

#### Data analysis

Firstly, we used descriptive statistics to examine participants’ characteristics. This study conducted a one-way analysis of variance (ANOVA) with SPSS version 26 to assess whether there were any significant differences between PSD, life satisfaction, and PMH.

**FIGURE 1:**
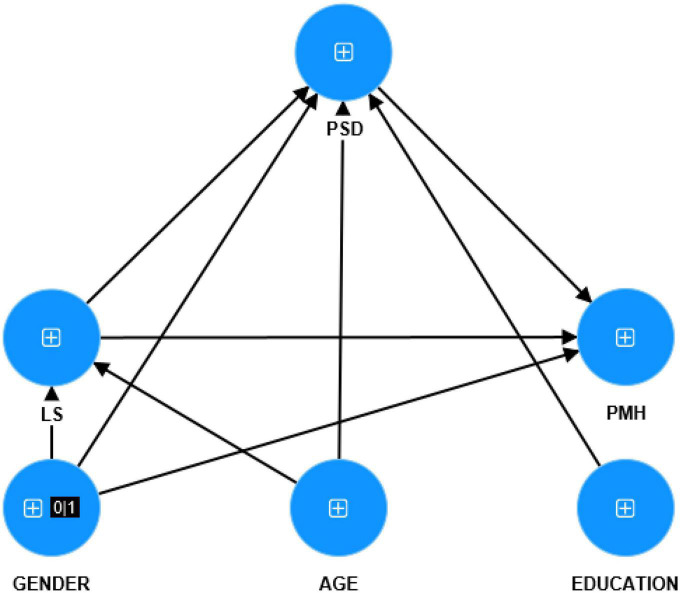
Hypothetical model. LS, life satisfaction; PSD, post-series depression; PMH, positive mental health.

The achieved data in this study was analyzed using smart partial least squares (SmartPLS)-SEM analysis, a variance-based structural equation modeling (Rigdon et al., 2017) in the latest release of SmartPLS 4 (4.0). Because of reflective measurement models, multiple independent-dependent relationships, moderation, and mediation hypothesized relationships, and non-normal data, PLS-SEM is selected to analyze the data (Hair et al., 2019). The systematic procedure for applying the PLS-SEM approach involves the assessment of the measurement model and the structural model. To evaluate the measurement model, reflective indicator reliability (outer loading), construct reliability [Cronbach’s alpha (CA), composite reliability], convergent validity (average variance extracted), and discriminant validity heterotrait-monotrait (HTMT criterion) were examined. To evaluate the structural model, collinearity statistics variance inflation factor (VIF), determination of coefficient (*R*^2^), the effect size f^2^ and the significance and relevance of path coefficients. Complete PLS-SEM analysis established on 1000 bootstrap samples was used to compute path coefficients with *P*-values and specific indirect, specific direct, and total effects. Although 5000 sample bootstrapping is typically used, our study only used 1,000 sample procedures due to our study’s sample size (*n* = 2,572). We conducted a multiple-mediated PLS path model with gender and education as single indicator input variables and PMH as output variable with PSD and LS mediating the effect of the input on the output variables. Hair et al. (2016) suggested prioritizing controlling the moderation effect before further analysis. Since that the moderator variable and independent variable should not principally be related, we alternatively accounted Age as the moderator (view Supplementary material) and independent variable (Figure 1) in the model.

## Results

### Measurement model

A Confirmatory Factor Analysis was performed using the PLS-SEM approach to assess the study’s measurement model. The common factor model was assessed involving indicator reliability, internal consistency reliability, convergent validity, and discriminant validity through outer loading values, Cronbach’s alpha, composite reliability, AVE, and HTMT criterion, respectively (Hair et al., 2019).

### Indicator reliability

The indicator reliability indicates the communality of an indicator, which is the degree to which endogenous constructs describe the indicator in a model (Avkiran, 2018). A such extent can be examined through the outer loading of an item. Outer loading or indicator loading is the bivariate correlation between indicator and construct (Hair et al., 2021). By squaring this index, it can be used to demonstrate the number of indicator variances explained by the host construct. To prove the reliability of the indicator, the first condition is that the indicator loading must be statistically significant. Then, Hair et al. (2021) highly recommend that indicator loadings greater than 0.708 represent acceptable indicator reliability since its square root implies that the construct explains more than half of the variance in the indicator. However, indicators with outer loadings ranging from 0.4 to 0.7 are still approved with some conditions involving preserving or reducing AVE and composite reliability (CR) values if the items are deleted (Hair et al., 2021). In Table 2, PMH2, PMH3, PMH6, PSD5, PSD7, PSD8, PSD14, and PSD15 were accepted as their outer loadings exceeded the recommended level of 0.7. All the remaining indicators’ outer loading below the 0.7 threshold surpassed the 0.4 level except for PSD1 being 0.379. Because the elimination of these indicators did not improve CR and AVE, they are conditionally accepted for the constructs that are the hosts of the indicators. All indicator loadings were significant at the 2.5% level with 1000 bootstrapping resampling procedures.

**TABLE 2:** Results of the reflective measurement model.

Construct and items	Loadings	CR	α	AVE
Life satisfaction (LS)	–	0.767	0.625	0.397
LS1	0.681	–	–	–
LS2	0.618	–	–	–
LS3	0.622	–	–	–
LS4	0.636	–	–	–
LS5	0.59	–	–	–
Positive mental health (PMH)	–	0.891	0.863	0.479
PMH1	0.623	–	–	–
PMH2	0.771	–	–	–
PMH3	0.745	–	–	–
PMH4	0.687	–	–	–
PMH5	0.696	–	–	–
PMH6	0.803	–	–	–
PMH7	0.661	–	–	–
PMH8	0.652	–	–	–
PMH9	0.56	–	–	–
Post-series depression (PSD)	–	0.902	0.884	0.388
PSD1	0.379	–	–	–
PSD2	0.619	–	–	–
PSD3	0.405	–	–	–
PSD4	0.622	–	–	–
PSD5	0.714	–	–	–
PSD6	0.628	–	–	–
PSD7	0.716	–	–	–
PSD8	0.741	–	–	–
PSD9	0.678	–	–	–
PSD10	0.512	–	–	–
PSD11	0.65	–	–	–
PSD12	0.527	–	–	–
PSD13	0.533	–	–	–
PSD14	0.748	–	–	–
PSD15	0.712	–	–	–

CR, composite reliability; AVE, average variance extracted.

### Construct reliability

The CA of the item is the most commonly relied on to measure internal consistency reliability. A major limitation of Cronbach’s alpha is that it assumes all indicator loadings are the same in the population (also referred to as tau-equivalence) (Hair et al., 2021). CR, therefore, can be more reliable as it weighs the differences in indicator loadings (Hair et al., 2019). However, CA is a rather conservative measure of reliability (i.e., it leads to relatively low reliability values) compared to CR, which tends to overestimate internal consistency reliability (i.e., leads to relatively higher reliability estimates). Therefore, the construct’s true reliability is typically viewed as within these two extreme values (Hair et al., 2021).

Both CA and CR have the same threshold: reliability values between 0.60 and 0.70 are considered “acceptable in exploratory research,” whereas values between 0.70 and 0.90 range from “satisfactory to good” values above 0.90 (and definitely above 0.95) are problematic since they indicate that the indicators are redundant, thereby reducing construct validity.

The results of CA and CR of our measurement model are presented in Table 2.

### Convergent validity

Average variance extracted is used to evaluate the convergent validity of measurements. The minimum acceptable AVE is 0.50—an AVE of 0.50 or higher indicates the construct explains 50 percent or more of the indicators’ variance that makes up the construct (Hair et al., 2019, 2021). However, in the case that CR is higher than 0.6, then AVE is less than 0.5 is still adequate (Fornell and Larcker, 1981). In Table 2, none of the measured constructs have an AVE value that surpasses the minimum threshold, but since all of their CR values are above 0.6, then the limitation of AVE is acceptable.

### Discriminant validity

Besides two commonly used methods to evaluate the discriminant value of the scale, which are Fornell and Lacker (1981) criterion and Cross factor loading, the HTMT criterion is an advanced method recently proposed by Dijkstra and Henseler (2015) and widely used. Hair et al. (2021) proposed that if the HTMT value of each pairwise construct does not exceed the 0.9 threshold, then the reflective model’s discriminability is validated. Next, a bootstrapping procedure is conducted to test whether the HTMT statistic is significantly different from 1. By that, a confidence interval range not including the value one can prove that two constructs are distinct (Hair et al., 2021).

The HTMT values are depicted in Table 3 along with the HTMT confidence interval is shown through the values in parentheses. All these values are below the maximum level of acceptance, with the exception HTMT value being 0.361 (LS and PMH). Confidence interval of all values do not include value of 0. The discriminant validity of the model, therefore, was established.

**TABLE 3:** Heterotrait-monotrait (HTMT) criterion.

	LS	PMH	PSD
**LS**	–	–	–
PMH	0.361 (0.305; 0.417)	–	–
PSD	0.204 (0.146; 0.249)	0.323 (0.278; 0.367)	–

PSD, post-series depression; LS, life satisfaction; PMH, positive mental health.

### Assessment of structural model

#### Collinearity statistics (VIF)

Variance inflation factor values of variables are measured to quantify the severity of collinearity issue of the structural model. Collinearity arises when two indicators are highly correlated. Table 4 reported that all VIF values are less than 10. The VIF values depicted no issue of collinearity in this achieved data.

**TABLE 4:** Collinearity statistics variance inflation factor (VIF).

	LS	MH	PSD
AGE	1.003	–	5.675
Education	–	–	5.651
Gender	1.003	1.025	1.024
LS	–	1.045	1.020
MH	–	–	–
PSD	–	1.035	–

PSD, post-series depression; LS, life satisfaction; PMH, positive mental health.

#### Determination of coefficient (*R*^2^)

*R*^2^ is a measure of the model’s predictive accuracy. Evaluation of the coefficients of determination represents the portion of variances in the endogenous constructs explained by the structural model. The value of *R*^2^ should be higher than 0.1 (Chin, 1998), which is considerable (Falk and Miller, 1992). This study found that 14.9% variance occurred in mental health explained by life satisfaction and PSD.

#### The effect sizes f^2^

Assessment of effect size allows the researcher to observe the effect of each exogenous construct on the endogenous construct. The values of f^2^ in this study are within the suggested level by Cohen (2013). LS to PMH, LS to PSD, and PSD to PMH have small effects of 0.06, 0.03, and 0.08, respectively.

#### Mediation analysis

The results demonstrated that the direct effect from LS to PMH (β = 0.231, *p* < 0.001) was positive and statistically significant, and PSD to PMH (β = –0.269, *p* < 0.001) was negative and statistically significant. This recent study had partial mediation owing to the significance of both direct and indirect effects (Nitzl et al., 2016).

### Results of PLS-SEM analysis

Figure 1 showed the final PLS model. The proposed research model for this study includes three different latent vectors: LS (comprising items from the Satisfaction with life scale), PSD (comprising items from the Post-series depression scale), and PMH (comprising items from Positive Mental Health Scale). In the mediation model, LS and PSD mediated the effect of GENDER, AGE, and EDUCATION (entered as single indicator input variables) on PMH latent vector.

We found that the model explained 14.9% of the variance in the PMH with both direct and indirect effects of variables. Results based on 1000 bootstrapped samples depicted a presentation of direct effects from PSD, LS, and Gender, along with indirect effects from Age, Education, and LS. The result from the Table 5 supported Hypothesis 1 as it revealed a positive effect of LS [β = 0.231, *p* < 0.001, 95% CI = (0.193; 0.273)] on PMH, while PSD [β = –0.269, *p* < 0.001, 95% CI = (–0.311; –0.229)] stated the negative effect (Hypothesis 3 supported). Furthermore, Hypothesis 2 was confirmed as an negative impact of LS on PSD [β = –0.165, *p* < 0.001, 95% CI = (–0.21; –0.125)] was reported. All direct effects were statistically significant at the 2.5% level, and the value of 0 was not included in the 95% confidence intervals.

**TABLE 5:** Results of structural model: Direct effects and indirect effects.

Path	β coefficient	*t*	*p*	95% confidence intervals	95% BC confidence intervals
**Direct effects**					
*AGE → LS*	0.059	2.904	0.004	(0.02; 0.1)	(0.017; 0.096)
*AGE → PSD*	–0.179	3.737	<0.001	(-0.273; -0.089)	(-0.272; -0.089)
*EDUCATION → PSD*	0.159	3.431	0.001	(0.069; 0.252)	(0.069; 0.252)
*GENDER → LS*	0.126	6.338	<0.001	(0.087; 0.162)	(0.087; 0.162)
*GENDER → PMH*	0.056	2.766	0.006	(0.016; 0.093)	(0.018; 0.098)
*GENDER → PSD*	0.09	4.499	<0.001	(0.049; 0.13)	(0.047; 0.125)
*LS → PMH*	0.231	10.944	<0.001	(0.193; 0.273)	(0.187; 0.27)
*LS → PSD*	–0.165	7.853	<0.001	(-0.21; -0.125)	(-0.202; -0.119)
*PSD → PMH*	–0.269	13.38	<0.001	(-0.311; -0.229)	(-0.304; -0.226)
**Indirect effects**					
*AGE → LS → PSD → PMH*	0.003	2.589	0.01	(0.001; 0.005)	(0.001; 0.005)
*LS → PSD → PMH*	0.044	6.94	<0.001	(0.033; 0.058)	(0.032; 0.057)
*Gender → LS → PSD → PMH*	0.006	4.656	<0.001	(0.004; 0.008)	(0.004; 0.008)
*AGE → LS → PMH*	0.014	2.752	0.006	(0.004; 0.024)	(0.004; 0.023)
*GENDER → LS → PMH*	0.029	5.463	<0.001	(0.019; 0.04)	(0.019; 0.04)
*EDUCATION → PSD → PMH*	–0.043	3.316	0.001	(-0.069; -0.019)	(-0.07; -0.019)
*AGE → PSD → PMH*	0.048	3.632	<0.001	(0.023; 0.0735	(0.023; 0.075)
*AGE → LS → PSD*	–0.01	2.656	0.008	(-0.018; -0.003)	(-0.017; -0.003)
*GENDER → PSD → PMH*	–0.024	4.239	<0.001	(-0.036; -0.013)	(-0.035; -0.013)
*GENDER → LS → PSD*	–0.021	4.759	<0.001	(-0.03; 0.014)	(-0.03; -0.013)

PSD, post-series depression; LS, life satisfaction; PMH, positive mental health; BC, bias corrected.

### Moderation and mediation analysis

Firstly, the moderation effect of Age on the relationship between LS and PMH was examined. When interpreting the results of a moderation analysis, the primary interest is with the significance of the interaction term (i.e., Age × LS to PMH). The result (see Supplementary Table 1) showed that Age did not moderate the relationship between PSD and PMH (*p* = 0.555 > 0.05) as well as the relationship between LS and PMH (*p* = 0.470 > 0.05). We then operated Age as an independent variable in our model.

All meditators are recommended to be considered in one model and analyzed simultaneously (Hair et al., 2021). Thus, we used a multi-step, multiple mediated PLS path model, with LS as input variables and PMH as output variables, while PSD mediated the effects of the input on the output variables, followed by the procedure described by Nitzl et al. (2016) to determine the mediation role of variables. Table 5 illustrated the specific indirect effects in the structural model.

The indirect effect of LS [β = 0.044, *p* < 0.001, 95% CI = (0.033; 0.058)] on PMH through PSD is significant, therefore, Hypothesis 4 is supported. Moreover, LS was earlier proven to have a positive direct impact on PMH (Table 4) which confirmed the complementary partial meditation role of PSD.

The results also revealed the presence of an indirect effect [β = 0.003, *p* = 0.01, 95% CI = (0.001; 0.005)] from Age to PMH with the contribution from LS and PSD, while the direct effect was not yielded. Moreover, the indirect path from Age orderly went through LS and PSD was significant. As a result, a serial causal model of LS and PSD fully mediated the relationship between Age and PMH was proposed. Our study also found significant indirect effects mediating by PSD from Education [β = –0.043, *p* = 0.001, 95% CI = (–0.069; 0.019)] and Gender [β = –0.024, *p* < 0.001, 95% CI = (–0.036; –0.013)] to PMH. Therefore, Hypotheses 5 and 6 are supported.

## Discussion

Using the PLS-SEM model, we aimed to investigate the direct and indirect effects of life satisfaction and PSD on individuals’ PMH. This is a novel and valuable contribution to understanding and promoting PMH, including emotional, psychological, and social wellbeing for good mental health (Keyes, 2002), and indicates positive functioning (Lukat et al., 2016) for both research and practice.

Life satisfaction is frequently studied as an outcome measure instead of a predictor for other variables (Coffey et al., 2015). Likewise, it has not been investigated whether life satisfaction can predict PSD and PMH over time. The findings showed that life satisfaction emerged as the strongest factor influencing the other constructs, including PSD and PMH. Firstly, we found that life satisfaction positively affected PMH. When seen life satisfaction has a positive effect, this relationship is backed up by numerous previous studies which concluded that positive emotions or effects would promote individuals’ PMH (Coffey et al., 2015; Bieda et al., 2019). This result also supported the broaden-and-build theory of Fredrickson (2001), which assumed that positive emotions could widen people’s momentary thought-action repertoires, decrease or eliminate negative emotions and improve individuals’ psychological wellbeing for a better life in the future. The evaluation of life satisfaction appeared to be based primarily on satisfaction in individuals’ important life domains, such as academic achievement, quality of romantic relationships, and family relationships which were related to a wide range of individuals’ social relations and mental wellbeing. However, several previous studies mentioned and suggested other factors that could affect individuals’ PMH, such as sleep quality (Peach et al., 2016), personality traits (Marino et al., 2018), flourishing, and quality of life (Singh and Junnarkar, 2015). Additionally, Kottasz et al. (2019) revealed that emptiness and nostalgia could influence the individuals’ level of PSD. The participants’ PMH could be affected by not only life satisfaction but also the simultaneous combination of those variables, especially in the COVID-19 context. This could explain the relatively small effect of life satisfaction on PMH in the study. Future research could further consider and conceptualize other models to investigate the determinants of PMH. The results obtained here may have implications for understanding the significant contributing role of positive emotions and their effects on individuals’ PMH when they are satisfied with their life.

As the data indicate, life satisfaction had a negative effect on PSD. Specifically, suppose individuals had a high level of life satisfaction. In that case, they could control and minimize unpleasant emotions (e.g., emptiness, melancholia, disappointment) triggered after their favorite screen products, such as movies and TV series, were discontinued. As mentioned above, the relationship between life satisfaction and PSD remained completely unexplored until now, but a growing body of empirical evidence suggested that the level of life satisfaction was considered an important indicator of protecting individuals’ mental health because it was negatively associated with loneliness (de Guzman et al., 2012), depressive symptoms (Bowlin and Baer, 2012) and the recurrence of substance use disorder (Laudet et al., 2009). Therefore, from a broader perspective, there are numerous scientific researches that supported the finding that the higher level of life satisfaction individuals had, the fewer negative emotions they suffered when finishing their much-loved films and TV series.

The negative relation between PSD and PMH has been investigated in the study. Specifically, PSD had a negative impact on PMH. This result illustrates that the higher levels of PSD people experience, the less likely they are to have PMH. Kottasz et al. (2019) defined PSD as negative feelings appearing when movies or TV series ended and PMH or mental wellbeing, which is a crucial component that should be examined in order to ensure mental health (Lukat et al., 2016). Based on the aforementioned definitions, this could be explained why PSD affected negatively PMH. Particularly, in the severe COVID-19 context, people were lacking in means and forms of entertainment. Consequently, deep in watched TV series or movies led to negative feelings such as sadness, disappointment, or regret when the movies or TV series ended. Moreover, Karlin (2015) also had a similar statement that PSD related to several negative emotions like sadness when the story was over, but people did not want it complete. People with PSD also had feelings of nostalgia and emptiness (Kottasz et al., 2019), which negatively impacted PMH.

Furthermore, the analysis also identified the role of PSD as a mediator between the relationships of LS and PMH. The influence of life satisfaction on PMH was partially mediated by PSD. Thus, individuals who were satisfied with life would have a higher level of PMH, although the negative effect of PSD diminished the longitudinal influence on PMH. It can be interpreted that PSD, which is characterized by negative emotions such as emptiness, boredom, and depression could lead to the reduction of individuals’ wellbeing by inhibiting the positive effects of life satisfaction on PMH. This achieved result is in accordance with several studies highlighting the negative impacts of those emotions on mental health. Shi et al. (2020) surveyed 917 Chinese people and reported that the degree of boredom could lead to a higher level of depression, anxiety, and stress in the context of the COVID-19 pandemic. A previous study by Newman and Sachs (2020) indicated that feelings of nostalgia had negative influences on wellbeing and these impacts were stronger when individuals felt more loneliness. Impliedly, it is suggested that PSD can be both entertaining and a risk factor that would negatively affect individuals’ PMH and lead to significant impairment to the ability to function effectively in daily life (Kottasz et al., 2019).

### Implications

The present findings offer theoretical and practical implications. Although the estimated effect of life satisfaction on PMH and PSD we discovered was relatively small, this finding contributed important evidence of associations between these variables. To our knowledge, this study is the first to empirically investigate the potential influence of PSD on individuals’ PMH in a psychological context. The results contribute an important document and clearer understanding of the negative impacts of problematic watching TV series or movies on mental health which is demonstrated in previous studies (Tefertiller and Maxwell, 2018). During the outbreak of the COVID-19 pandemic, lots of people suffered deleterious effects, which led to the worsening of mental health, such as feelings of depression and anxiety. The problematic watching screen products in leisure time or when individuals take restrictive measures such as lockdowns, social distancing, and voluntary self-isolation would cause severe impacts on mental health instead of reducing stress and boosting mood. In this regard, family members and friends need to contact continuously and communicate to assess, identify, and prevent individuals from wellbeing problems, negative changes, and outcomes (Ellis et al., 2020; Espinoza and Hernandez, 2022) engagement in TV series or movies such as emptiness, loneliness, and depression. Spending more time with family members or high levels of friend communication could support and protect individuals from excessive engagement in TV series or movies which would trigger negative feelings such as emptiness, loneliness, and depression (Ellis et al., 2020; Espinoza and Hernandez, 2022). Supporting wellbeing problems and promoting mental health for people have to be a high priority during the pandemic and post-pandemic. This research has provided novel evidence and document on the effects of PSD on mental health which make a premise for further consistent studies on PSD with other aspects of psychological research. Psychology researchers will be able to approach this concept in a new context and open up numerous interesting scientific ideas for research into PSD in many other scientific disciplines. The relationship between PSD and PMH which is reported in could be valuable evidence and document for further study focusing on the factors leading to the reduction in psychological wellbeing.

Our findings also provide additional information about the association between life satisfaction and the aspects of mental health, especially PMH. Mental health promotion is not merely about studying adverse psychological symptoms and distress or clinical intervention, but also factors strengthening personal resources and complete mental health. Cognitive interventions to enhance life satisfaction will also lead to increased positive affect later, which are prevention of significant psychological distress and associated positively with higher mental health. The authors suggest a dire need to communicate information and educate people about the palpable effects of excessive engagement in TV series or movies as a viewing habit that could cause deleterious effects on psychological wellbeing. Improving understanding of PSD to avoid its negative influences has become pertinent at this time, owing to essential information and knowledge to protect their mental health. Besides, the authors hope that the achieved results provide valuable and useful evidence for designing and improving psychological interventions aiming at enhancing individuals’ wellbeing. Psychological therapies and clinical intervention should focus on behavioral strategies such as effectively managing time, focusing on the positive, taking responsibility for actions, pursuing meaningful tasks, experiences, and attitudes, re-evaluating relationships, creating social networks for increased social support and savoring the joys of life for individuals which would induce and develop positive emotions. Those strategies could assist people in recognizing their personal resources, nurture and improve the feeling of satisfaction with life and prevent the risk factors for better wellbeing because positive emotions can enhance emotional wellbeing, improve problem-solving attitude and reduce negative thinking (Joiner et al., 2001).

### Limitation

This study is the first study to demonstrate the theoretical relationship between PSD and life satisfaction and PMH which suggests several new research directions and research interests for future research. Nevertheless, there are limitations that should be acknowledged. This cross-sectional study could not demonstrate the longitudinal effects of life satisfaction and PSD on PMH. Because of the phase of the lockdown period from September 2021 to December 2021, the cross-sectional study is the most appropriate design in research method to generate data rapidly and infer some health-related events or for generating hypotheses on the topic (Sedgwick, 2014). Future studies should design an experimental or longitudinal study to determine the influences of those factors on individuals’ wellbeing and detect potential factors which could affect and lead to other interesting findings because a high level of life satisfaction is important but not sufficient for a complete mental health, especially PMH. PSD and PMH were self-reported which could bias reporting. Further study should apply other measures or data collection methods such as interviews. There is a need for additional research to replicate and extend our reports by controlling potential confounding variables to allow more accurate evaluation of relationships between factors and robust conclusions.

## Conclusion

Our cross-sectional study provided the first and important evidence about the influences of life satisfaction and PSD on PMH in the Vietnamese population. The results showed (i) a positive relationship between life satisfaction and PMH when life satisfaction acted as an outcome measure instead of a predictor; (ii) a negative relationship between life satisfaction and PSD; (iii) a negative association between PSD and PMH; and (iv) the influence of life satisfaction on PMH was partially mediated by PSD. The findings contribute an important document and clearer understanding of the negative impacts of problematic watching TV series or movies on mental health. Besides, additional information about the association between life satisfaction and the aspects of mental health, especially PMH is also provided and highlighted. The authors suggest a dire need to communicate information and educate people about the palpable effects of excessive engagement in TV series or movies as a viewing habit that could cause deleterious effects on psychological wellbeing. This is the first study on not only PSD in psychological context, but also the effects of life satisfaction and PSD on PMH among Vietnamese participants, contributing interesting findings and research directions for further studies.

## Data availability statement

The raw data supporting the conclusions of this article will be made available by the authors, without undue reservation.

## Ethics statement

The studies involving human participants were reviewed and approved by the Declaration of Helsinki and the ethical principles of the American Psychological Association (APA) regarding research involving human participants. The patients/participants provided their written informed consent to participate in this study.

## Author contributions

V-LT-C contributed to conception and design of the study. T-TN-T, T-NN, and B-TN-D organized the database and wrote the first draft of the manuscript. T-TN-T and T-NN performed the statistical analysis. All authors contributed to manuscript revision, read, and approved the submitted version.
