# Supplementary material for: Mediation effects of post-series depression on the relationship between life satisfaction and positive mental health of Vietnamese: A cross-sectional study in COVID-19 pandemic context
Source: Front Psychol. 2022 Nov 28;13:971711. doi: 10.3389/fpsyg.2022.971711 (PMC9744194; doi:10.3389/fpsyg.2022.971711)
Supplement: Supplementary file 1 [file Data_Sheet_1.pdf]

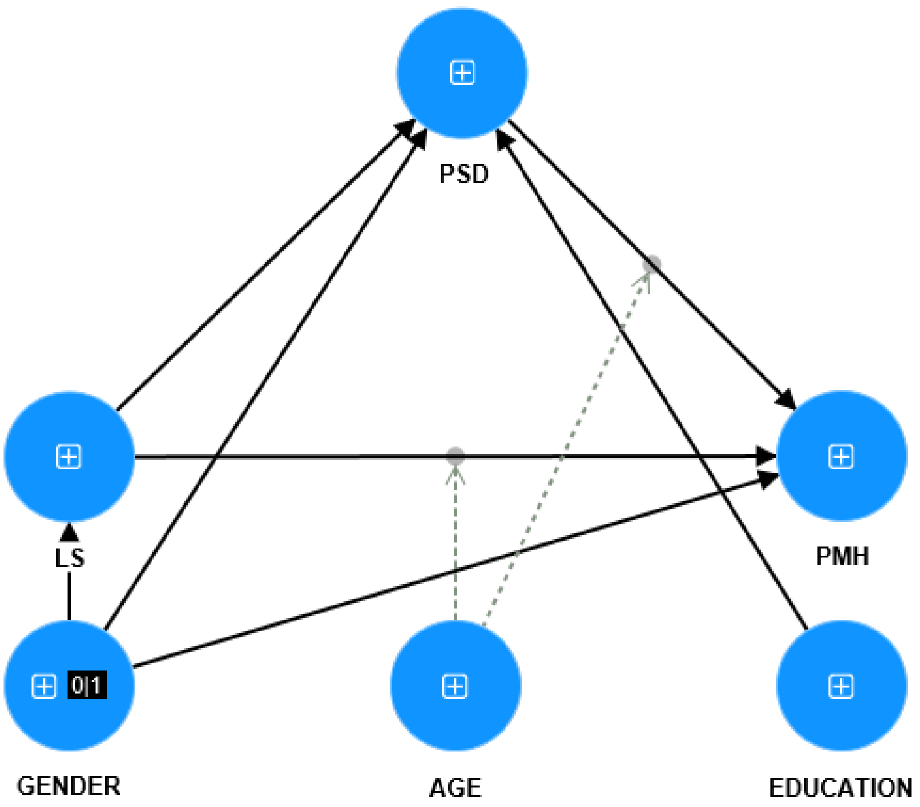


**Figure 1.** *Hypothetical model. Age operated as a moderator.*

**Table 1.** *Results of structural model: Age operated as a moderator.*

| *Path* | *β coefficient* | *T Statistics* | *P values* |
|---|---|---|---|
| *AGE → PMH* | *0.031* | *1.705* | *0.088* |
| *EDUCATION → PSD* | *-0.001* | *0.022* | *0.982* |
| *GENDER → LS* | *0.284* | *5.921* | *0.000* |
| *GENDER → PMH* | *0.064* | *1.501* | *0.134* |
| *GENDER → PSD* | *0.238* | *4.975* | *0.000* |
| *LS → PMH* | *0.462* | *25.455* | *0.000* |
| *LS → PSD* | *-0.201* | *9.992* | *0.000* |
| *PSD → PMH* | *-0.215* | *11.284* | *0.000* |
| *AGE x PSD → PMH* | *-0.011* | *0.590* | *0.555* |
| *AGE x LS → PMH* | *0.014* | 0.722 | 0.470 |
